# Supplementary material for: OpenMM 7: Rapid development of high performance algorithms for molecular dynamics
Source: PLoS Comput Biol. 2017 Jul 26;13(7):e1005659. doi: 10.1371/journal.pcbi.1005659 (PMC5549999; doi:10.1371/journal.pcbi.1005659)
Supplement: S1 Source Code — (ZIP) [file pcbi.1005659.s002.zip › OpenMM-7.0.1-Source/docs/api-python/generated/simtk.openmm.openmm.CustomNonbondedForce.html]

CustomNonbondedForce — OpenMM 7.0.1.dev-5e86c4f documentation


# CustomNonbondedForce¶

*class* `simtk.openmm.openmm.``CustomNonbondedForce`(*\*args*)¶
:   This class implements nonbonded interactions between particles. Unlike NonbondedForce, the functional form of the interaction is completely customizable, and may involve arbitrary algebraic expressions and tabulated functions. It may depend on the distance between particles, as well as on arbitrary global and per-particle parameters. It also optionally supports periodic boundary conditions and cutoffs for long range interactions.

    To use this class, create a CustomNonbondedForce object, passing an algebraic expression to the constructor that defines the interaction energy between each pair of particles. The expression may depend on r, the distance between the particles, as well as on any parameters you choose. Then call addPerParticleParameter() to define per-particle parameters, and addGlobalParameter() to define global parameters. The values of per-particle parameters are specified as part of the system definition, while values of global parameters may be modified during a simulation by calling Context::setParameter().

    Next, call addParticle() once for each particle in the System to set the values of its per-particle parameters. The number of particles for which you set parameters must be exactly equal to the number of particles in the System, or else an exception will be thrown when you try to create a Context. After a particle has been added, you can modify its parameters by calling setParticleParameters(). This will have no effect on Contexts that already exist unless you call updateParametersInContext().

    CustomNonbondedForce also lets you specify “exclusions”, particular pairs of particles whose interactions should be omitted from force and energy calculations. This is most often used for particles that are bonded to each other.

    As an example, the following code creates a CustomNonbondedForce that implements a 12-6 Lennard-Jones potential:

    ```
    CustomNonbondedForce* force = new CustomNonbondedForce("4*epsilon*((sigma/r)^12-(sigma/r)^6); sigma=0.5*(sigma1+sigma2); epsilon=sqrt(epsilon1*epsilon2)");
    ```

    This force depends on two parameters: sigma and epsilon. The following code defines these as per-particle parameters:

    ```
    force->addPerParticleParameter("sigma");
    force->addPerParticleParameter("epsilon");
    ```

    The expression \_must\_ be symmetric with respect to the two particles. It typically will only be evaluated once for each pair of particles, and no guarantee is made about which particle will be identified as “particle 1”. In the above example, the energy only depends on the products sigma1\*sigma2 and epsilon1\*epsilon2, both of which are unchanged if the labels 1 and 2 are reversed. In contrast, if it depended on the difference sigma1-sigma2, the results would be undefined, because reversing the labels 1 and 2 would change the energy.

    CustomNonbondedForce can operate in two modes. By default, it computes the interaction of every particle in the System with every other particle. Alternatively, you can restrict it to only a subset of particle pairs. To do this, specify one or more “interaction groups”. An interaction group consists of two sets of particles that should interact with each other. Every particle in the first set interacts with every particle in the second set. For example, you might use this feature to compute a solute-solvent interaction energy, while omitting all interactions between two solute atoms or two solvent atoms.

    To create an interaction group, call addInteractionGroup(). You may add as many interaction groups as you want. Be aware of the following:

    > - Exclusions are still taken into account, so the interactions between excluded pairs are omitted.
    > - Likewise, a particle will never interact with itself, even if it appears in both sets of an interaction group.
    > - If a particle pair appears in two different interaction groups, its interaction will be computed twice. This is sometimes useful, but be aware of it so you do not accidentally create unwanted duplicate interactions.
    > - If you do not add any interaction groups to a CustomNonbondedForce, it operates in the default mode where every particle interacts with every other particle.

    When using a cutoff, by default the interaction is sharply truncated at the cutoff distance. Optionally you can instead use a switching function to make the interaction smoothly go to zero over a finite distance range. To enable this, call setUseSwitchingFunction(). You must also call setSwitchingDistance() to specify the distance at which the interaction should begin to decrease. The switching distance must be less than the cutoff distance. Of course, you could also incorporate the switching function directly into your energy expression, but there are several advantages to keeping it separate. It makes your energy expression simpler to write and understand. It allows you to use the same energy expression with or without a cutoff. Also, when using a long range correction (see below), separating out the switching function allows the correction to be calculated more accurately.

    Another optional feature of this class is to add a contribution to the energy which approximates the effect of all interactions beyond the cutoff in a periodic system. When running a simulation at constant pressure, this can improve the quality of the result. Call setUseLongRangeCorrection() to enable it.

    Computing the long range correction takes negligible work in each time step, but it does require an expensive precomputation at the start of the simulation. Furthermore, that precomputation must be repeated every time a global parameter changes (or when you modify per-particle parameters by calling updateParametersInContext()). This means that if parameters change frequently, the long range correction can be very slow. For this reason, it is disabled by default.

    Expressions may involve the operators + (add), - (subtract), \* (multiply), / (divide), and ^ (power), and the following functions: sqrt, exp, log, sin, cos, sec, csc, tan, cot, asin, acos, atan, sinh, cosh, tanh, erf, erfc, min, max, abs, floor, ceil, step, delta, select. All trigonometric functions are defined in radians, and log is the natural logarithm. step(x) = 0 if x is less than 0, 1 otherwise. delta(x) = 1 if x is 0, 0 otherwise. select(x,y,z) = z if x = 0, y otherwise. The names of per-particle parameters have the suffix “1” or “2” appended to them to indicate the values for the two interacting particles. As seen in the above example, the expression may also involve intermediate quantities that are defined following the main expression, using ”;” as a separator.

    In addition, you can call addTabulatedFunction() to define a new function based on tabulated values. You specify the function by creating a TabulatedFunction object. That function can then appear in the expression.

    `__init__`(*self*, *energy*) → CustomNonbondedForce¶
    :   \_\_init\_\_(self, rhs) -> CustomNonbondedForce

        Create a CustomNonbondedForce.

        |  |  |
        | --- | --- |
        | Parameters: | **energy** (*string*) – an algebraic expression giving the interaction energy between two particles as a function of r, the distance between them, as well as any global and per-particle parameters |

    Methods

    |  |  |
    | --- | --- |
    | `__init__`((self, energy) -> CustomNonbondedForce) | \_\_init\_\_(self, rhs) -> CustomNonbondedForce |
    | `addExclusion`((self, particle1, particle2) -> int) | Add a particle pair to the list of interactions that should be excluded. |
    | `addFunction`((self, name, values, min, ...) | Add a tabulated function that may appear in the energy expression. |
    | `addGlobalParameter`((self, name, ...) | Add a new global parameter that the interaction may depend on. |
    | `addInteractionGroup`((self, set1, set2) -> int) | Add an interaction group. |
    | `addParticle`((self, parameters) -> int) | addParticle(self) -> int |
    | `addPerParticleParameter`((self, name) -> int) | Add a new per-particle parameter that the interaction may depend on. |
    | `addTabulatedFunction`((self, name, ...) | Add a tabulated function that may appear in the energy expression. |
    | `createExclusionsFromBonds`(self, bonds, ...) | Identify exclusions based on the molecular topology. |
    | `getCutoffDistance`((self) -> double) | Get the cutoff distance (in nm) being used for nonbonded interactions. |
    | `getEnergyFunction`((self) -> std::string const &) | Get the algebraic expression that gives the interaction energy between two particles |
    | `getExclusionParticles`(self, index) | Get the particles in a pair whose interaction should be excluded. |
    | `getForceGroup`((self) -> int) | Get the force group this Force belongs to. |
    | `getFunctionParameters`(self, index) | Get the parameters for a tabulated function that may appear in the energy expression. |
    | `getGlobalParameterDefaultValue`((self, ...) | Get the default value of a global parameter. |
    | `getGlobalParameterName`((self, ...) | Get the name of a global parameter. |
    | `getInteractionGroupParameters`(self, index) | Get the parameters for an interaction group. |
    | `getNonbondedMethod`(...) | Get the method used for handling long range nonbonded interactions. |
    | `getNumExclusions`((self) -> int) | Get the number of particle pairs whose interactions should be excluded. |
    | `getNumFunctions`((self) -> int) | Get the number of tabulated functions that have been defined. |
    | `getNumGlobalParameters`((self) -> int) | Get the number of global parameters that the interaction depends on. |
    | `getNumInteractionGroups`((self) -> int) | Get the number of interaction groups that have been defined. |
    | `getNumParticles`((self) -> int) | Get the number of particles for which force field parameters have been defined. |
    | `getNumPerParticleParameters`((self) -> int) | Get the number of per-particle parameters that the interaction depends on. |
    | `getNumTabulatedFunctions`((self) -> int) | Get the number of tabulated functions that have been defined. |
    | `getParticleParameters`(self, index) | Get the nonbonded force parameters for a particle. |
    | `getPerParticleParameterName`((self, ...) | Get the name of a per-particle parameter. |
    | `getSwitchingDistance`((self) -> double) | Get the distance at which the switching function begins to reduce the interaction. |
    | `getTabulatedFunction`((self, ...) | getTabulatedFunction(self, index) -> TabulatedFunction |
    | `getTabulatedFunctionName`((self, ...) | Get the name of a tabulated function that may appear in the energy expression. |
    | `getUseLongRangeCorrection`((self) -> bool) | Get whether to add a correction to the energy to compensate for the cutoff and switching function. |
    | `getUseSwitchingFunction`((self) -> bool) | Get whether a switching function is applied to the interaction. |
    | `setCutoffDistance`(self, distance) | Set the cutoff distance (in nm) being used for nonbonded interactions. |
    | `setEnergyFunction`(self, energy) | Set the algebraic expression that gives the interaction energy between two particles |
    | `setExclusionParticles`(self, index, ...) | Set the particles in a pair whose interaction should be excluded. |
    | `setForceGroup`(self, group) | Set the force group this Force belongs to. |
    | `setFunctionParameters`(self, index, name, ...) | Set the parameters for a tabulated function that may appear in the energy expression. |
    | `setGlobalParameterDefaultValue`(self, index, ...) | Set the default value of a global parameter. |
    | `setGlobalParameterName`(self, index, name) | Set the name of a global parameter. |
    | `setInteractionGroupParameters`(self, index, ...) | Set the parameters for an interaction group. |
    | `setNonbondedMethod`(self, method) | Set the method used for handling long range nonbonded interactions. |
    | `setParticleParameters`(self, index, parameters) | Set the nonbonded force parameters for a particle. |
    | `setPerParticleParameterName`(self, index, name) | Set the name of a per-particle parameter. |
    | `setSwitchingDistance`(self, distance) | Set the distance at which the switching function begins to reduce the interaction. |
    | `setUseLongRangeCorrection`(self, use) | Set whether to add a correction to the energy to compensate for the cutoff and switching function. |
    | `setUseSwitchingFunction`(self, use) | Set whether a switching function is applied to the interaction. |
    | `updateParametersInContext`(self, context) | Update the per-particle parameters in a Context to match those stored in this Force object. |
    | `usesPeriodicBoundaryConditions`((self) -> bool) | Returns whether or not this force makes use of periodic boundary conditions. |

    Attributes

    |  |  |
    | --- | --- |
    | `CutoffNonPeriodic` |  |
    | `CutoffPeriodic` |  |
    | `NoCutoff` |  |

    `getNumParticles`(*self*) → int¶
    :   Get the number of particles for which force field parameters have been defined.

    `getNumExclusions`(*self*) → int¶
    :   Get the number of particle pairs whose interactions should be excluded.

    `getNumPerParticleParameters`(*self*) → int¶
    :   Get the number of per-particle parameters that the interaction depends on.

    `getNumGlobalParameters`(*self*) → int¶
    :   Get the number of global parameters that the interaction depends on.

    `getNumTabulatedFunctions`(*self*) → int¶
    :   Get the number of tabulated functions that have been defined.

    `getNumFunctions`(*self*) → int¶
    :   Get the number of tabulated functions that have been defined.

    `getNumInteractionGroups`(*self*) → int¶
    :   Get the number of interaction groups that have been defined.

    `getEnergyFunction`(*self*) → std::string const &¶
    :   Get the algebraic expression that gives the interaction energy between two particles

    `setEnergyFunction`(*self*, *energy*)¶
    :   Set the algebraic expression that gives the interaction energy between two particles

    `getNonbondedMethod`(*self*) → OpenMM::CustomNonbondedForce::NonbondedMethod¶
    :   Get the method used for handling long range nonbonded interactions.

    `setNonbondedMethod`(*self*, *method*)¶
    :   Set the method used for handling long range nonbonded interactions.

    `getCutoffDistance`(*self*) → double¶
    :   Get the cutoff distance (in nm) being used for nonbonded interactions. If the NonbondedMethod in use is NoCutoff, this value will have no effect.

        |  |  |
        | --- | --- |
        | Returns: | the cutoff distance, measured in nm |
        | Return type: | double |

    `setCutoffDistance`(*self*, *distance*)¶
    :   Set the cutoff distance (in nm) being used for nonbonded interactions. If the NonbondedMethod in use is NoCutoff, this value will have no effect.

        |  |  |
        | --- | --- |
        | Parameters: | **distance** (*double*) – the cutoff distance, measured in nm |

    `getUseSwitchingFunction`(*self*) → bool¶
    :   Get whether a switching function is applied to the interaction. If the nonbonded method is set to NoCutoff, this option is ignored.

    `setUseSwitchingFunction`(*self*, *use*)¶
    :   Set whether a switching function is applied to the interaction. If the nonbonded method is set to NoCutoff, this option is ignored.

    `getSwitchingDistance`(*self*) → double¶
    :   Get the distance at which the switching function begins to reduce the interaction. This must be less than the cutoff distance.

    `setSwitchingDistance`(*self*, *distance*)¶
    :   Set the distance at which the switching function begins to reduce the interaction. This must be less than the cutoff distance.

    `getUseLongRangeCorrection`(*self*) → bool¶
    :   Get whether to add a correction to the energy to compensate for the cutoff and switching function. This has no effect if periodic boundary conditions are not used.

    `setUseLongRangeCorrection`(*self*, *use*)¶
    :   Set whether to add a correction to the energy to compensate for the cutoff and switching function. This has no effect if periodic boundary conditions are not used.

    `addPerParticleParameter`(*self*, *name*) → int¶
    :   Add a new per-particle parameter that the interaction may depend on.

        |  |  |
        | --- | --- |
        | Parameters: | **name** (*string*) – the name of the parameter |
        | Returns: | the index of the parameter that was added |
        | Return type: | int |

    `getPerParticleParameterName`(*self*, *index*) → std::string const &¶
    :   Get the name of a per-particle parameter.

        |  |  |
        | --- | --- |
        | Parameters: | **index** (*int*) – the index of the parameter for which to get the name |
        | Returns: | the parameter name |
        | Return type: | string |

    `setPerParticleParameterName`(*self*, *index*, *name*)¶
    :   Set the name of a per-particle parameter.

        |  |  |
        | --- | --- |
        | Parameters: | - **index** (*int*) – the index of the parameter for which to set the name - **name** (*string*) – the name of the parameter |

    `addGlobalParameter`(*self*, *name*, *defaultValue*) → int¶
    :   Add a new global parameter that the interaction may depend on.

        |  |  |
        | --- | --- |
        | Parameters: | - **name** (*string*) – the name of the parameter - **defaultValue** (*double*) – the default value of the parameter |
        | Returns: | the index of the parameter that was added |
        | Return type: | int |

    `getGlobalParameterName`(*self*, *index*) → std::string const &¶
    :   Get the name of a global parameter.

        |  |  |
        | --- | --- |
        | Parameters: | **index** (*int*) – the index of the parameter for which to get the name |
        | Returns: | the parameter name |
        | Return type: | string |

    `setGlobalParameterName`(*self*, *index*, *name*)¶
    :   Set the name of a global parameter.

        |  |  |
        | --- | --- |
        | Parameters: | - **index** (*int*) – the index of the parameter for which to set the name - **name** (*string*) – the name of the parameter |

    `getGlobalParameterDefaultValue`(*self*, *index*) → double¶
    :   Get the default value of a global parameter.

        |  |  |
        | --- | --- |
        | Parameters: | **index** (*int*) – the index of the parameter for which to get the default value |
        | Returns: | the parameter default value |
        | Return type: | double |

    `setGlobalParameterDefaultValue`(*self*, *index*, *defaultValue*)¶
    :   Set the default value of a global parameter.

        |  |  |
        | --- | --- |
        | Parameters: | - **index** (*int*) – the index of the parameter for which to set the default value - **defaultValue** (*double*) – the default value of the parameter |

    `addParticle`(*self*, *parameters*) → int¶
    :   addParticle(self) -> int

        Add the nonbonded force parameters for a particle. This should be called once for each particle in the System. When it is called for the i’th time, it specifies the parameters for the i’th particle.

        |  |  |
        | --- | --- |
        | Parameters: | **parameters** (*vector< double >*) – the list of parameters for the new particle |
        | Returns: | the index of the particle that was added |
        | Return type: | int |

    `getParticleParameters`(*self*, *index*)¶
    :   Get the nonbonded force parameters for a particle.

        |  |  |
        | --- | --- |
        | Parameters: | **index** (*int*) – the index of the particle for which to get parameters |
        | Returns: | **parameters** – the list of parameters for the specified particle |
        | Return type: | vector< double > |

    `setParticleParameters`(*self*, *index*, *parameters*)¶
    :   Set the nonbonded force parameters for a particle.

        |  |  |
        | --- | --- |
        | Parameters: | - **index** (*int*) – the index of the particle for which to set parameters - **parameters** (*vector< double >*) – the list of parameters for the specified particle |

    `addExclusion`(*self*, *particle1*, *particle2*) → int¶
    :   Add a particle pair to the list of interactions that should be excluded.

        In many cases, you can use createExclusionsFromBonds() rather than adding each exclusion explicitly.

        |  |  |
        | --- | --- |
        | Parameters: | - **particle1** (*int*) – the index of the first particle in the pair - **particle2** (*int*) – the index of the second particle in the pair |
        | Returns: | the index of the exclusion that was added |
        | Return type: | int |

    `getExclusionParticles`(*self*, *index*)¶
    :   Get the particles in a pair whose interaction should be excluded.

        |  |  |
        | --- | --- |
        | Parameters: | **index** (*int*) – the index of the exclusion for which to get particle indices |
        | Returns: | - **particle1** (*int*) –   the index of the first particle in the pair - **particle2** (*int*) –   the index of the second particle in the pair |

    `setExclusionParticles`(*self*, *index*, *particle1*, *particle2*)¶
    :   Set the particles in a pair whose interaction should be excluded.

        |  |  |
        | --- | --- |
        | Parameters: | - **index** (*int*) – the index of the exclusion for which to set particle indices - **particle1** (*int*) – the index of the first particle in the pair - **particle2** (*int*) – the index of the second particle in the pair |

    `createExclusionsFromBonds`(*self*, *bonds*, *bondCutoff*)¶
    :   Identify exclusions based on the molecular topology. Particles which are separated by up to a specified number of bonds are added as exclusions.

        |  |  |
        | --- | --- |
        | Parameters: | - **bonds** (*vector< std::pair< int, int > >*) – the set of bonds based on which to construct exclusions. Each element specifies the indices of two particles that are bonded to each other. - **bondCutoff** (*int*) – pairs of particles that are separated by this many bonds or fewer are added to the list of exclusions |

    `addTabulatedFunction`(*self*, *name*, *function*) → int¶
    :   Add a tabulated function that may appear in the energy expression.

        |  |  |
        | --- | --- |
        | Parameters: | - **name** (*string*) – the name of the function as it appears in expressions - **function** (*TabulatedFunction \**) – a TabulatedFunction object defining the function. The TabulatedFunction should have been created on the heap with the “new” operator. The Force takes over ownership of it, and deletes it when the Force itself is deleted. |
        | Returns: | the index of the function that was added |
        | Return type: | int |

    `getTabulatedFunction`(*self*, *index*) → TabulatedFunction¶
    :   getTabulatedFunction(self, index) -> TabulatedFunction

        Get a reference to a tabulated function that may appear in the energy expression.

        |  |  |
        | --- | --- |
        | Parameters: | **index** (*int*) – the index of the function to get |
        | Returns: | the TabulatedFunction object defining the function |
        | Return type: | TabulatedFunction |

    `getTabulatedFunctionName`(*self*, *index*) → std::string const &¶
    :   Get the name of a tabulated function that may appear in the energy expression.

        |  |  |
        | --- | --- |
        | Parameters: | **index** (*int*) – the index of the function to get |
        | Returns: | the name of the function as it appears in expressions |
        | Return type: | string |

    `addFunction`(*self*, *name*, *values*, *min*, *max*) → int¶
    :   Add a tabulated function that may appear in the energy expression.

    `getFunctionParameters`(*self*, *index*)¶
    :   Get the parameters for a tabulated function that may appear in the energy expression.

    `setFunctionParameters`(*self*, *index*, *name*, *values*, *min*, *max*)¶
    :   Set the parameters for a tabulated function that may appear in the energy expression.

    `addInteractionGroup`(*self*, *set1*, *set2*) → int¶
    :   Add an interaction group. An interaction will be computed between every particle in set1 and every particle in set2.

        |  |  |
        | --- | --- |
        | Parameters: | - **set1** (*set< int >*) – the first set of particles forming the interaction group - **set2** (*set< int >*) – the second set of particles forming the interaction group |
        | Returns: | the index of the interaction group that was added |
        | Return type: | int |

    `getInteractionGroupParameters`(*self*, *index*)¶
    :   Get the parameters for an interaction group.

        |  |  |
        | --- | --- |
        | Parameters: | **index** (*int*) – the index of the interaction group for which to get parameters |
        | Returns: | - **set1** (*set< int >*) –   the first set of particles forming the interaction group - **set2** (*set< int >*) –   the second set of particles forming the interaction group |

    `setInteractionGroupParameters`(*self*, *index*, *set1*, *set2*)¶
    :   Set the parameters for an interaction group.

        |  |  |
        | --- | --- |
        | Parameters: | - **index** (*int*) – the index of the interaction group for which to set parameters - **set1** (*set< int >*) – the first set of particles forming the interaction group - **set2** (*set< int >*) – the second set of particles forming the interaction group |

    `updateParametersInContext`(*self*, *context*)¶
    :   Update the per-particle parameters in a Context to match those stored in this Force object. This method provides an efficient method to update certain parameters in an existing Context without needing to reinitialize it. Simply call setParticleParameters() to modify this object’s parameters, then call updateParametersInContext() to copy them over to the Context.

        This method has several limitations. The only information it updates is the values of per-particle parameters. All other aspects of the Force (the energy function, nonbonded method, cutoff distance, etc.) are unaffected and can only be changed by reinitializing the Context. Also, this method cannot be used to add new particles, only to change the parameters of existing ones.

    `usesPeriodicBoundaryConditions`(*self*) → bool¶
    :   Returns whether or not this force makes use of periodic boundary conditions.

        |  |  |
        | --- | --- |
        | Returns: | true if force uses PBC and false otherwise |
        | Return type: | bool |

    `__delattr__`¶
    :   x.\_\_delattr\_\_(‘name’) <==> del x.name

    `__format__`()¶
    :   default object formatter

    `__getattribute__`¶
    :   x.\_\_getattribute\_\_(‘name’) <==> x.name

    `__hash__`¶

    `__reduce__`()¶
    :   helper for pickle

    `__reduce_ex__`()¶
    :   helper for pickle

    `__sizeof__`() → int¶
    :   size of object in memory, in bytes

    `__str__`¶

    `getForceGroup`(*self*) → int¶
    :   Get the force group this Force belongs to.

    `setForceGroup`(*self*, *group*)¶
    :   Set the force group this Force belongs to.

        |  |  |
        | --- | --- |
        | Parameters: | **group** (*int*) – the group index. Legal values are between 0 and 31 (inclusive). |

High performance molecular simulation on GPUs


### Navigation

- Application Layer
- Library Layer
  - Core Objects
  - Forces
  - Integrators
  - Extras

©2015, Stanford University and the Authors.
|
Powered by Sphinx 1.3.1
& Alabaster 0.7.7
|
Page source
